# Prioritizing HIV/AIDS prevention strategies in Bandung, Indonesia: A cost analysis of three different HIV/AIDS interventions

**DOI:** 10.1371/journal.pone.0221078

**Published:** 2019-08-15

**Authors:** Inge de Bresser, Toine E. P. Remers, Monse W. M. Wieland, Rozar Prawiranegara, Adiatma Y. M. Siregar, Rob Baltussen

**Affiliations:** 1 Department for Health Evidence, Radboud University Medical Center, Nijmegen, The Netherlands; 2 Department of Economics, Padjadjaran University, Badung, Indonesia; University of Ghana College of Health Sciences, GHANA

## Abstract

**Background:**

Indonesia has one of the fastest growing HIV epidemics in Asia, which mainly concentrates within risk groups. Several strategies are available to combat this epidemic, like outreach to Men who have Sex with Men (MSM) and transgender, Harm Reduction Community Meetings (HRCMs) for Injecting Drug Users (IDUs), and Information, Education and Communication (IEC) programs at Maternal & Child Health Posts (MCHPs). Reliable cost data are currently not present, hampering HIV/AIDS priority setting. The aim of this study thus is to assess the societal costs of outreach programs to MSM and transgender, HRCMs for IDUs and IEC at MCHPs in Bandung, Indonesia in 2016.

**Methods:**

The societal costs were collected in Bandung from April until May 2017. Health care costs were collected by interviewing stakeholders, using a micro-costing approach. Non-health care costs were determined by conducting surveys within the target groups of the interventions.

**Results:**

The societal costs of the outreach program were US$ 347,199.03 in 2016 and US$ 73.72 per reached individual. Moreover, the cost of HRCM for IDUs were US$ 48,618.31 in 2016 and US$ 365.55 per community meeting. For the IEC program at MCHPs, US$ 337.13 was paid in 2016 and the cost per visitor were US$ 0.51.

**Conclusion:**

This study provides valuable insights in the costs of outreach to MSM and transgender, HRCMs for IDUs and IEC at MCHPs. Policy makers can use these results in setting priorities within Indonesia. Data on effectiveness of interventions is necessary to make conclusive statements regarding cost-effectiveness and priority of interventions.

## Introduction

With one of the fastest growing incidence rates in Asia and a total of 690,000 people living with HIV, HIV is a big problem in Indonesia [1–3]. Despite a relatively low overall prevalence (0.27%), the prevalence among HIV risk-groups is much higher, reaching up to 25.8% among Men who have Sex with Men (MSM), 24.8% among transgender people, and 28.8% among injecting drug users (IDU) [4]. Both lack of knowledge on one hand, and stigma towards people living with HIV and their relatives on the other hand are amongst the main causes of the growing epidemic [5, 6].

Several strategies are available to bring a halt to the HIV epidemic in Indonesia, including outreach, harm-reduction community meetings (HRCMs) and Information, Education and Communication (IEC) programs. Outreach focuses on reaching hidden populations of HIV risk groups to engage them in the process of reducing HIV risk behaviors [7]. HRCMs aim at increasing knowledge and awareness about several HIV related topics among IDUs. IEC is a program developed to reduce stigma and improve knowledge in several population groups, like mothers and pregnant women.

Although many HIV/AIDS interventions are available, not all of them can be implemented. In fact, when implementing all HIV interventions described in the strategy of Indonesia in 2014, an amount of US$ 208 million would have been required, whereas only US$ 97 million was available [8, 9]. This US$ 111 million funding gap emphasizes the need for prioritizing strategies in this country. Multi-Criteria Decision Analysis (MCDA) is a method to support such priority setting in resource-limited settings with optimal use of available resources [10].

One important criterion considered in an MCDA, is cost-effectiveness. Determining this criterion requires comprehensive and reliable data on the costs of implementing HIV/AIDS interventions. This type of data is however scarce and highly dependent on the specific type and setting of a program. Thus, a reliable cost analysis of all available HIV/AIDS interventions in Indonesia is needed. This study therefore aims to assess the societal costs of outreach programs to MSM and transgenders, harm-reduction community meetings for IDUs, and information, education and communication programs at maternal and child health posts in Bandung, Indonesia in 2016.

## Materials and methods

### Study setting and population

This study was conducted in Bandung, the capital of West Java province. The HIV/AIDS epidemic that Bandung is facing is a concentrated epidemic comparable to the national estimate [11].

#### Outreach to MSM and TG

The outreach to both MSM and TG living in Bandung is conducted by a non-governmental organization (NGO) in Bandung, called Srikandi Pasundan (SP). The outreach program of SP is focussing on informing these key populations about HIV/AIDS and referring them to HIV testing facilities. The program is performed 26 times a month by both paid workers and volunteers, who visit HIV hotspots like malls, parks, and gyms and have a chat, hand out information packages, or refer individuals at risk to HIV testing facilities.

#### Harm reduction community meetings for IDUs

The HRCMs in Bandung are organized by a NGO called Grapiks for IDUs from around Bandung with the main goal of increasing IDU’s knowledge about the effects of drug use. Several topics are discussed, including HIV/AIDS, tuberculosis, hepatitis, legal issues of drug use, and sterilization of needles. The meetings are provided by staff members of Grapiks and community health centres (the so-called puskesmas) at either community-health centres (11 times a month) or HIV/AIDS clinics (once a month).

#### IEC at maternal & child health posts

Maternal & child health posts (MCHPs) (the so-called posyandus) are monthly-organized health facilities in Indonesia, where pregnant women or mothers with their children can come for a health check. Besides health measurement, the MHCP also offers IEC about a wide range of health topics, among which HIV/AIDS. The HIV/AIDS IEC program consists of a monthly IEC stand and an IEC presentation, which is organized three to four times a year. The program is coordinated by Warga Peduli AIDS (WPA), a civil society organization concerned with HIV/AIDS, with each district having one or more WPA volunteers who provide the program.

### Data collection and cost estimation

Data collection took place in April and May 2017 and focused on the costs made in 2016. Costs were estimated from a societal perspective according the World Health Organization’s (WHO) guidelines for cost analysis in primary health care [12]. We made a distinction between health care costs and non-health care costs. Costs were collected in Indonesian Rupiah (IDR) and converted to US$ using the official 2016 annual conversion rate [8]. Data were both registered and analysed in Microsoft Office Excel 2007. A detailed description of collection and estimation of costs can be found elsewhere [13–15].

Data on health care costs were collected by interviewing stakeholders involved in the program, being staff from the coordinating organization (four employees from Grapiks, the program manager and one of the fieldworkers from Srikandi Pasundan, and four employees from the MCHP’s) or staff from the Bandung Aids Commission (KPA Bandung). Written informed-consent was obtained prior to all interviews with these stakeholders and participants received a reimbursement of IDR 100.000 for their participation afterwards. Health-care costs were further divided into capital costs and recurrent costs. Capital costs were defined as costs incurred for resources that last longer than one year. Recurrent costs were defined as costs incurred for resources that are purchased regularly. All inputs related to the programs were identified, classified, and quantified using a micro-costing approach [16].

Capital costs were estimated annually with a discount rate of 3% [16]. Annual costs were based on the working life of the capital resource and the costs of purchasing that resource in 2016. Working life of buildings was assumed to be twenty years, of trainings to be ten years and of furniture and equipment to be five years, based on general agreements. Purchase costs were based on documented data, market prices or expert opinion alternatively.

Recurrent costs were calculated by multiplying the costs of a resource unit by the yearly quantity of usage of the resource unit. Data on quantity of usage were based on documented data or expert opinion alternatively. Costs of a resource unit were based on documented data, salary registers, market prices or expert opinion.

Household costs were the only included non-health care costs and were further divided into productivity loss costs and travel costs. Productivity loss costs were defined as the income that the visitors miss because of spending their time at the program and were based on the value of their leisure time. Data on these subjects were obtained with a survey conducted among 16 MSM and 13 TG of the outreach program, 23 IDUs visiting the HRCMs and 35 visitors of the MCHPs (see S1 Appendix: survey questionnaires). Verbal informed consent was obtained from all participants prior to filling in the questionnaire and participants received a small reimbursement in the form of a pack of biscuits afterwards. The survey contained questions about monthly income, daily working hours, monthly expenditure, travel time, and travel costs. We determined productivity loss costs by measuring the amount of time people spent attending a program and multiplying this by the value of their leisure time. For employed visitors, the leisure time value was calculated based on their self-reported monthly salary, whereas non-employed visitors were assumed to have a leisure time value equal to the minimum salary.

Additionally, various attendants of the IEC presentation indicated a desire to increase the frequency of presentations. A scale-up scenario analysis was thus performed determining the annual societal costs if the IEC presentation would be scaled up, i.e. providing it every month instead of three to four times a year.

### Sensitivity analysis

As several cost calculations relied on assumptions, a sensitivity analysis was performed to determine the impact of variable uncertainty on the societal costs. The analysis was performed on the biggest assumptions (i.e. underlying the largest cost categories of each intervention) with a plausible 30% uncertainty range being applied. Assumptions included for outreach were duration of meetings, number of referrals and distance travelled, whereas expert opinion and duration of meetings were included for HRCMs. For the IEC program, assumptions on the value of leisure time of WPA volunteers and unemployed visitors were analysed.

## Results

In 2016, the SP outreach program reached 4500 MSM and 210 TG, resulting in a total number of 4710 people being reached. When looking at HRCMs, a total number of 133 meetings were conducted in 11 community-health centres and one HIV/AIDS clinic. The HIV/AIDS IEC stand was visited by 8 people a month and each presentation was visited by 22 people on average. The average yearly number of visitors per MCHP of both the stand and the presentation was 641.

The societal costs paid in 2016 for all programs and the share of total costs for all categories are shown in Table 1. Outreach was the most expensive program with a total of US$ 347,199, followed by HRCM costing US$ 48,618 and IEC being the least expensive program with US$ 337 in total societal costs.

**Table 1:** Societal costs (in US$) and cost distribution profile (in % of societal costs) of outreach to Men who have Sex with Men and Transgender, harm reduction community meetings for Internal Drug Users, and Information, Education and Communication programs at Maternal and Child Health Posts in 2016 in Bandung, Indonesia.

Type of cost
	Outreach	%	HRCM	%	IEC	%
**HEALTH CARE**						
Capital (annual)						
Building and furniture	1,019.57	0.3	184.39	0.4	10.19	3.0
Training	-	-	-	-	4.60	2.2
Equipment	262.91	0.1	414.08	0.9	0.05	0.0
Recurrent						
Personnel	111,718.51	32.2	13,509.59	27.8	88.97	26.4
Training	6,834.42	2.0	1,445.81	3.0	33.38	11.4
Equipment	-	-	504.95	1.0	0.28	0.1
Supplies	12,621.58	3.6	4,224.47	8.7	51.10	15.2
Transport	16,594.59	4.8	14,990.61	30.8	-	-
Building: maintenance	1,082.03	0.3	2,925.44	6.0	-	-
Short-term consultancies	2,051.35	0.6	-	-	-	-
**Sub-total health care**	**152,184.97**	**43.8**	**38,797.80**	**79.8**	**193.58**	**57.4**
**NON-HEALTH CARE**						
Productivity loss	153,995.43	46.8	10,418.98	21.4	134.08	39.8
Two-way travel costs	32,413.75	9.3	5,100.39^1^	10.5	9.47	0.03
**Sub-total non-health care**	**195,014.06**	**56.2**	**15,519.36**	**31.9**	**143.55**	**42.6**
**Societal costs**	**347,199.03**	**100**	**48,618.30**	**100**	**337.13**	**100**

HRCM = Harm Reducation Community Meeting, IEC = Information, Education, and Communication

^1^:Two-way travel costs were not included in the societal costs since IDUs were compensated with transportation fees.

The costs for reaching out to one individual every week during the entire year of 2016, were US$ 73.72. HRCMs costed US$ 365.55 per meeting and the societal costs for providing IEC to one visitor were US$ 0.51. Non-health care costs accounted for the biggest share in total costs in all three programs, followed by personnel costs for outreach and IEC, and by transport cost for the HRCMs.

The additional upscale scenario of the IEC program at MCHPs showed that when intensifying the frequency of the presentation from three to four times a year to 12 times a year, the annual societal costs of the program will become US$ 788.76, which is US$ 451.63 more than in 2016. Moreover, the societal costs paid for providing the IEC program to one visitor, will become US$ 0.47, compared to US$ 0.51 in 2016.

A 15% over- or underestimation in duration of outreach resulted in a deviation of US$ 5.18 (7.0%) in reaching out to one individual and US$ 24,396 in societal costs. Both number of referrals and distance travelled seemed to have no significant effects on costs. For HRCMs, a deviation of 15% in values estimated by experts resulted in an in- or decrease of societal costs with US$ 4,275.00 (8.8%). A 15% and 30% decrease of the duration of HRCMs resulted in a decrease of the societal cost of respectively US$ 3,589 (7.4%) and US$ 7,179 (14.8%). In the societal costs of IEC, a 15% over- and underestimation of the value of leisure time of unemployed volunteers and visitors resulted in a US$ 18.88 and US$ 0.03 deviation of total costs and social costs per visitor respectively.

## Discussion

### Main findings

This study examined the societal costs of outreach to MSM and TG, harm reduction community meetings for IDUs and information, education and communication programs at maternal and child health posts in 2016. The societal costs for the interventions in 2016 were US$ 347,199, US$ 48,618 and US$ 337.13 respectively. Besides, this study discovered that the costs for reaching out to one MSM or TG for one year were US$ 73.72. For organizing one harm reduction community meeting, US$ 365.55 had to be paid. Besides, the costs for providing IEC to one visitor of the MCHP were US$ 0.51.

For both the outreach- and IEC program, the second largest cost category was personnel. Despite being a big cost item of the SP outreach program, saving on personnel costs should be done with caution as it might reduce the effectiveness of the intervention. Cutting on personnel would likely result in outreach workers being less able to really take time for their clients and by means of that affect the special bond between workers and clients. Saving costs of personnel, or in fact any cost category of IEC, does not seem very preferable as costs are already low and savings could hamper effectiveness.

Transport costs could be a more suitable category for cutting costs in both outreach and HRCMs. These costs are mostly determined by transportation fees given to either outreach workers or participants of HRCMs. These fees are often higher than the transport costs that are actually made. Adjusting this system of transportation fees in a way that fees approximate the actual money spent, seems therefore a reasonable way of cutting costs. For the HRCMs however, reducing this transportation fee might make the HRCMs less attractive for the IDUs to visit and might therefore reduce the effectiveness of the program. Savings should thus be done with caution.

Productivity loss costs formed a large component of the overall societal costs in all three interventions, eliciting an ongoing economic debate on the value of leisure time. In order to gain a comprehensive societal cost overview, we chose to base visitors’ leisure time values on market wage rates. Although this decision largely determined the productivity loss costs, the financial burden on visitors should not be neglected when adopting a societal perspective.

The low annual frequency combined with the expressed desire by IEC visitors to increase the intensity of the IEC program, raised questions about the costs of intensifying the program. Upscaling IEC resulted in an extra amount of US$ 451.63 to be paid annually. However, when considering the costs per visitor, it was shown that the upscale scenario would be as efficient as current practice, paying US$ 0.47 per visitor compared to US$ 0.51 in current practice. Nevertheless, the question remains whether the increase in annual societal costs is affordable. Upscaling of outreach and HRCMs was deemed irrelevant, since these interventions were already implemented on a frequent basis and large scale.

### Limitations

Although the methods of our study were largely based on the guidelines as described in the WHO manual for cost analysis in primary health care [12], this study had some limitations.

First, some cost components were solely based on expert opinion as this was often the only way of calculating costs in absence of documented data. Prices that were the result from expert opinion were however checked with local market prices or follow-up interviews if possible and, for HRCMs, expert opinion was included in the sensitivity analyses to show influences on outcomes.

Secondly, the cost analysis of outreach and HRCMs were both conducted for one single organisation in Bandung, meaning conclusions on costs can only be drawn for this single organisation. It is however expected that the way outreach and HRCM activities are undertaken in Bandung is comparable to other regions. The results of this study might therefore be considered a rough cost estimate of other comparable programs in Indonesia. This does not apply to the IEC program as this was done for an organization which implements the program throughout the whole country. The small sample size of three MCHPs in this study however means extrapolation should be done with caution as well.

Thirdly, the non-health care costs calculations were based on questionnaires filled in by 16 MSM and 13 TG, 23 IDUs, and 35 women visiting MCHPs meaning non-health care costs found in this study might not represent the health care costs of the entire population.

Lastly, assumptions needed to be made to complete the cost analysis. This might cause a deviation from the true costs if assumptions were not in line with reality. However, sensitivity analyses showed the influence of over- or underestimations of the biggest assumptions being made is low (<10%).

### Available literature & future research

No other cost analyses of these specific HIV/AIDS interventions in Indonesia have been done so far, hampering any comparison to other results in similar settings. A comparable micro-costing study in the African republic of Benin however reported the costs of outreach through peer educators to be 38,95 US$ per person reached [17]. These considerably lower costs of outreach in Benin compared to outreach for MSM and transgender in Indonesia are most likely attributable to the service provider’s perspective being chosen excluding non-health care costs, amounting >56% of total costs for outreach in this study, from the analysis. Additionally, the economic costs of IEC for the general public in Andhra Pradesh state, India, were measured to be 0.16 US$ per person. As these costs do not include non-health care costs either, these findings are comparable with our findings for the costs of IEC at MCHPs of 0.51 US$ per person [18].

More importantly however, only three out of the many available HIV/AIDS interventions in Bandung were included in this study [19]. Several other programs in Bandung have already been analysed and significant efforts are made to review all other programs, but a full overview is still lacking [20–23]. Additional micro-costing studies should thus be conducted until this full overview, ready to be used in an MCDA, is available.

MCDA however often considers cost-effectiveness instead of costs, meaning data on effectiveness of all interventions should be available as well. In contrast to cost data, there is hardly any data on effectiveness of interventions meaning studies determining the effectiveness of all interventions should be conducted as well as soon as possible.

Additionally, Siregar et al. showed that support of peers/family is important in avoiding loss-to-follow-up of HIV/AIDS patients [24]. Encouraging family members to support their relative might thus help to avoid a possible decrease in effectiveness caused by implementing savings in transportation fees for HRCMs.

Lastly, other studies support our finding that scaling up of a HIV/AIDS intervention within Bandung could prove to be a valuable step with low additional costs [25, 26].

## Conclusion

The total societal costs of outreach to MSM and Transgender, HRCMs to IDUs, and IEC at MCHPs are US$ 347,199, US$ 48,618, and US$337.13 respectively. It can be concluded that the IEC program is a low-cost program and that costs of the outreach program are relatively high with the HRCMs being not the most expensive nor the cheapest program.

### Recommendations

As this study was performed to inform the currently ongoing MCDA process in Bandung, our first recommendation is to combine our cost data with results on the effectiveness of the interventions and use them for this process. Similarly, cost and effectiveness data of other HIV/AIDS interventions in Bandung should be included in the MCDA as well.

Considering costs and the contribution of each cost category to the societal costs, a few recommendations can be made as well. First, it is recommended to critically examine and possibly revise the system of transportation fees for both outreach workers and participants of HRCMs. The effectiveness of both programs should however always be in scope to e.g. prevent the scenario in which less clients will come to HRCMs. As the costs of the IEC program are already relatively low, it is not recommended to reduce any cost category. In fact, we recommend to implement the upscale scenario as you reach more visitors and by means of that possibly increase the effectiveness of the program. Before doing so, it should however be investigated if any organization involved in the program is willing and/or able to fund the upscaling of IEC presentation.

## Supporting information

S1 Appendix Survey questionnaires. (DOCX)
