# Supplementary material for: Prioritizing HIV/AIDS prevention strategies in Bandung, Indonesia: A cost analysis of three different HIV/AIDS interventions
Source: PLoS One. 2019 Aug 15;14(8):e0221078. doi: 10.1371/journal.pone.0221078 (PMC6695116; doi:10.1371/journal.pone.0221078)
Supplement: S1 Appendix — (DOCX) [file pone.0221078.s001.docx]

# S1 appendix: Survey questionnaires

**Outreach to MSM and TG**

**Questionnaire**

**Please answer the following questions by writing down your answers, or by ticking (√) the appropriate box (). If you have trouble in answering a question, do not hesitate to ask the enumerator.**

| **A.** | **RESPONDENT PROFILE** | | |
| --- | --- | --- | --- |
| 1. | ID : | __________________________________________ | |
| 2. | Place of stay : | __________________________________________ | |
| 3. | Sex: | **•** Male 1 **•** Female 2 **•** Transgender 3 | |
| 4. | Age : | ………… year | |
| 5. | Last degree of completed education: | | |
|  |  1. Not going to school  2. Primary School  3. Junior High School  4. Senior High School   5. Diploma  6. Bachelor Degree  7. Master Degree  8. Doctoral Degree | | |
| 6. | Current marital status: | | |
|  |  not yet married 0  married 1  divorced 2  widowed 3 | | |
| 7. | Number of children | ………… | |
| **B** | **OCCUPATION AND INCOME** | | |
| 8. | What is your current occupation (may give more than one answer, proceed no 12 it non paid job) | | |
|  | ---------------------------------------------------------------------------------------------------------------------------- | | |
| 9. | What is your average monthly income?  (if you have more than one occupation, please state the total income)    **Rp** …………….…………………… | | |
| 10. | How many hours do you work per day? | | …… hours |
| 11. | How many days do you work per week? | | …… days |
| 12. | To fulfill your own monthly needs, do you also receive money from other people? Please state in the following table as well as the amount. | | |
|  | \| **No** \| **aReceive money from** \| **bLast month amount** \| \| --- \| --- \| --- \| \| **1** \| Parents \| Rp…………………………….. \| \| **2** \| Siblings \| Rp…………………………….. \| \| **3** \| Other relatives \| Rp…………………………….. \| \| **4** \| Children \| Rp…………………………….. \| \| **5** \| Friends \| Rp…………………………….. \| \| **6** \| Selling own goods \| Rp…………………………….. \| \| **7** \| Borrow from …. \| Rp…………………………….. \| \| **8** \| Others, please state………. \| Rp…………………………….. \| | | |
|  |  | | |
| 13. | Please state and detail your own monthly expenses | | |
|  | \| **No** \| **aExpenses** \| **bLast month amount** \| \| --- \| --- \| --- \| \| **1** \| House rent/mortgage \| Rp ………………………………………… \| \| **2** \| Electricity, water, telephone \| Rp ………………………………………… \| \| **3** \| Water \| Rp ………………………………………… \| \| **4** \| Telephone \| Rp ………………………………………… \| \| **5** \| Transport/gasoline \| Rp ………………………………………… \| \| **6** \| Cellphone credit \| Rp ………………………………………… \| \| **7** \| Food at home \| Rp ………………………………………… \| \| **8** \| Food out of home \| Rp ………………………………………… \| \| **9** \| Entertainment (i.e. snacks, cinema) \| Rp ………………………………………… \| \| **10** \| Cigarettes \| Rp ………………………………………… \| \| **11** \| Health/doctor fee \| Rp ………………………………………… \| \| **12** \| Medication \| Rp ………………………………………… \| \| **13** \| Savings \| Rp ………………………………………… \| \| **14** \| Others, please state……………………… \| Rp………………………………………….. \| | | |
|  |  | | |
| 14. | Who is currently staying with you?  **To fill the following tabel**:   - Please circle the number of the person who is currently living with you, if he/she is not on the list, please state on number 7-10 - Please state the occupation of the person (including housewife, students, or unemployed) and his/her income | | |
|  | \| 1. Family member/   accompanying person \| \| 1. Occupation \| 1. Monthly income \| 1. Does this person accompany you to the health facility? \| \| --- \| --- \| --- \| --- \| --- \| \| 1 \| Father \| ...... \| Rp........................ \| **** \| \| 2 \| Mother \| ...... \| Rp........................ \| **** \| \| 3 \| Older sibling \| ...... \| Rp........................ \| **** \| \| 4 \| Younger sibling \| ...... \| Rp........................ \| **** \| \| 5 \| Spouse \| ...... \| Rp........................ \| **** \| \| 6 \| Child \| ...... \| Rp........................ \| **** \| \| 7 \| ...... \| ...... \| Rp........................ \| **** \| \| 8 \| ...... \| ...... \| Rp........................ \| **** \| \| 9 \| ...... \| ...... \| Rp........................ \| **** \| \| 10 \| ...... \| ...... \| Rp........................ \| **** \| | | |

| 15. | How do you reach the facility? | |
| --- | --- | --- |
|  |  1. On foot   2. Bicycle   3. Motorcycle   4. Motorcycle taxi   5. Car |  6. Public transport (car)   7. Taxi   8. Public transport (bus)   9. Others, please state………… |
| 16. | How long is your travel time to reach the facility? | …… minutes |
| 17. | On your visit to the facility, how much do you spent in average for: | |
|  | \| 1. Two way transport for yourself \| Rp................ \| \| --- \| --- \| \| 1. Two way transport for person(s) accompanying you \| Rp................ \| \| 1. Registration \| Rp................ \| \| 1. Medical treatment \| Rp................ \| \| 1. Laboratory check \| Rp................ \| \| 1. Outreach meeting \| Rp................ \| \| 1. Materials received during meeting \| Rp................ \| \| 1. …… \| Rp................ \| | |

18. How much time do you spent in the facility? (including waiting time)……..minutes

19 Was this the first time you visited an outreach meeting?

- Yes
- No

**IF NO,** how often do you visit an outreach meeting?

**Please write down the total number of visits a week, month or year*

......................................................................................................................................................................................................................................................................................................................................

...................................................................................................................................................................

What mark ranging from 1 up to 10 would you give the outreach meeting in terms of clarity of the information that was give to you? Please explain.

......................................................................................................................................................................................................................................................................................................................................

...................................................................................................................................................................

What mark ranging from 1 up to 10 would you give the outreach meeting in terms of the skills of the outreach worker that conducts the outreach? Please explain.

......................................................................................................................................................................................................................................................................................................................................

...................................................................................................................................................................

What mark ranging from 1 up to 10 would you give the outreach meeting in terms of accessibility of the outreach meeting? Please explain.

......................................................................................................................................................................................................................................................................................................................................

...................................................................................................................................................................

Was the information that was given to you during the outreach meeting sufficient to know where to look at in terms of HIV/AIDS prevention and treatment?

- Yes
- No

**IF NO,** what else would you have liked to learn about this during the meeting?

......................................................................................................................................................................................................................................................................................................................................

...................................................................................................................................................................

What are positive aspects of the outreach meeting?

......................................................................................................................................................................................................................................................................................................................................

...................................................................................................................................................................

What are things that can be improved about the outreach meeting?

......................................................................................................................................................................................................................................................................................................................................

..................................................................................................................................................................

**Kuisioner**

**Jawab pertanyaan-pertanyaan di bawah ini dengan cara menuliskan jawaban, menceklis (√) di kotak (•). Apabila Anda punya pertanyaan, jangan ragu untuk bertanya kepada enumerator.**

| **A.** | **PROFIL RESPONDEN** | | |
| --- | --- | --- | --- |
| 1. | NAMA : | __________________________________________ | |
| 2. | Tempat Tinggal : | __________________________________________ | |
| 3. | Jenis Kelamin : | **•** Pria 1 **•** Wanita 2 **•** Transgender 3 | |
| 4. | Usia : | ………… tahun | |
| 5. | Pendidikan terakhir: | | |
|  | **•** 1. Tidak bersekolah **•** 2. Sekolah Dasar **•** 3. Sekolah Menengah Pertama **•** 4. Sekolah Menengah Atas **•** 5. Diploma **•** 6. Sarjana **•** 7. Master **•** 8. Doktor | | |
| 6. | Status Pernikahan: | | |
|  | **•** belum menikah 0 **•** menikah 1 **•** bercerai 2 **•** janda 3 | | |
| 7. | Jumlah anak | ………… | |
| **B** | **PEKERJAAN DAN PENDAPATAN** | | |
| 8. | Apa pekerjaan Anda saat ini? (boleh menjawab lebih dari satu pekerjaan, langsung ke pertanyaan nomor 12 apabila pekerjaan adalah pekerjaan tidak dibayar) | | |
|  | ---------------------------------------------------------------------------------------------------------------------------- | | |
| 9. | Berapa pendapatan rata-rata Anda perbulan? (apabila Anda mempunyai lebih dari satu pekerjaan, sebutkan pendapatan total)    **Rp** …………….…………………… | | |
| 10. | Berapa jam Anda bekerja setiap hari? | | …… jam |
| 11. | Berapa hari Anda bekerja setiap minggu? | | …… har |
| 12. | Untuk memenuhi kebutuhan Anda perbulan, apakah Anda menerima uang dari pihak lain? Sebutkan di table di bawah ini sesuai dengan jumlahnya. | | |
|  | \| **No** \| **Mendapatkan uang dari** \| **Jumlah bulan kemarin** \| \| --- \| --- \| --- \| \| **1** \| Orang Tua \| Rp…………………………….. \| \| **2** \| Kakak/Adik \| Rp…………………………….. \| \| **3** \| Saudara Lain \| Rp…………………………….. \| \| **4** \| Anak-anak \| Rp…………………………….. \| \| **5** \| Teman-teman \| Rp…………………………….. \| \| **6** \| Menjual barang pribadi \| Rp…………………………….. \| \| **7** \| Meminjam dari …. \| Rp…………………………….. \| \| **8** \| Lain-lain, sebutkan………. \| Rp…………………………….. \| | | |
|  |  | | |
| 13. | Sebutkan pengeluaran Anda setiap bulannya | | |
|  | \| **No** \| **Pengeluaran** \| **Jumlah bulan kemarin** \| \| --- \| --- \| --- \| \| **1** \| Sewa rumah/mortgage \| Rp ………………………………………… \| \| **2** \| Listrik \| Rp ………………………………………… \| \| **3** \| Air \| Rp ………………………………………… \| \| **4** \| Telepon \| Rp ………………………………………… \| \| **5** \| Transportasi/bensin \| Rp ………………………………………… \| \| **6** \| Pulsa \| Rp ………………………………………… \| \| **7** \| Makanan di rumah \| Rp ………………………………………… \| \| **8** \| Makanan di luar rumah \| Rp ………………………………………… \| \| **9** \| Hiburan (i.e. snacks, bioskop) \| Rp ………………………………………… \| \| **10** \| Rokok \| Rp ………………………………………… \| \| **11** \| Kesehatan \| Rp ………………………………………… \| \| **12** \| Obat-obatan \| Rp ………………………………………… \| \| **13** \| Tabungan \| Rp ………………………………………… \| \| **14** \| Lain-lain, sebutkan……………………… \| Rp………………………………………….. \| | | |
|  |  | | |
| 14. | Siapa yang saat ini tinggal bersama Anda?  **Untuk mengisi tabel berikut ini**:   - Bulatkan jumlah orang yang saat ini tinggal bersama Anda, jika dia tidak ada di daftar, sebutkan di nomor 7-10 - Sebutkan pekerjaan orang tersebut (termasuk ibu rumah tangga, pelajar, atau pengangguran) dan pendapatan mereka | | |
|  | \| 1. Anggota keluarga/   Orang yang tinggal bersama \| \| 1. Pekerjaan \| 1. Pendapatan perbulan \| 1. Apakah orang tersebut menemani Anda ke fasilitas kesehatan? \| \| --- \| --- \| --- \| --- \| --- \| \| 1 \| Ayah \| ...... \| Rp........................ \|  \| \| 2 \| Ibu \| ...... \| Rp........................ \|  \| \| 3 \| Kakak \| ...... \| Rp........................ \|  \| \| 4 \| Adik \| ...... \| Rp........................ \|  \| \| 5 \| Pasangan \| ...... \| Rp........................ \|  \| \| 6 \| Anak \| ...... \| Rp........................ \|  \| \| 7 \| ...... \| ...... \| Rp........................ \|  \| \| 8 \| ...... \| ...... \| Rp........................ \|  \| | | |

| 15. | Bagaimana Anda pergi ke fasilitas kesehatan? | |
| --- | --- | --- |
|  | **•** 1. Jalan kaki  **•** 2. Sepeda  **•** 3. Sepeda motor  **•** 4. Ojek  **•** 5. Mobil | **•** 6. Transportasi umum (mobil)  **•** 7. Taxi  **•** 8. Transportasi umum (bus)  **•** 9. Lain-lain,sebutkan………… |
| 16. | Berapa lama perjalanan Anda ke lokasi outreach? | …… menit |
| 17. | Dalam kunjungan Anda ke fasilitas kesehatan, berapa banyak pengeluaran rata-rata Anda untuk: | |
|  | \| 1. Transportasi pulang pergi untuk Anda sendiri \| Rp................ \| \| --- \| --- \| \| 1. Transportasi pulang pergi untuk orang-orang yang menemani Anda \| Rp................ \| \| 1. Rapat penyuluhan \| Rp................ \| \| 1. Item yang diterima di rapat \| Rp................ \| \| 1. …… \| Rp................ \| | |

18. Berapa lama waktu yang Anda habiskan di lokasi outreach? …… menit

19. Apakah ini pertama kalinya Anda menghadiri pertemuan outreach?

- Iya
- Tidak

**Jika Tidak,** seberapa sering Anda menghadiri pertemuan outreach?

**Tulis jumlah total kehadiran setiap minggu, bulan, atau tahun*

......................................................................................................................................................................................................................................................................................................................................

...................................................................................................................................................................

20. Dari 1 sampai 10, Berapa nilai yang akan Anda berikan kepada pertemuan outreach mengenai kejelasan informasi yang diberikan kepada Anda? Tolong jelaskan.

......................................................................................................................................................................................................................................................................................................................................

...................................................................................................................................................................

21. Dari 1 sampai 10, Berapa nilai yang akan Anda berikan kepada pertemuan outreach mengenai kemampuan pekerja penyuluhan yang melakukan penyuluhan? Tolong jelaskan.

......................................................................................................................................................................................................................................................................................................................................

...................................................................................................................................................................

22. Dari 1 sampai 10, Berapa nilai yang akan Anda berikan kepada pertemuan outreach mengenai aksesibilitas dari rapat penyuluhan? Tolong jelaskan.

......................................................................................................................................................................................................................................................................................................................................

...................................................................................................................................................................

23. Apakah informasi yang diberikan kepada Anda ketika pertemuan outreach cukup untuk mengetahui ke mana Anda harus mencari informasi tentang pencegahan dan pengobatan HIV/AIDS?

- Iya
- Tidak

**JIKA TIDAK,** Apa saja yang ingin Anda pelajari di pertemuan outreach?....................................................................................................................................................................................................................................................................................................................................

...................................................................................................................................................................

24. Apa aspek-aspek positif dari pertemuan outreach?

......................................................................................................................................................................................................................................................................................................................................

...................................................................................................................................................................

25. Apa saja yang bias ditingkatkan dari pertemuan outreach?

....................................................................................................................................................................................................................................................................................................................................................

..........................................................................................................................................................................

**Harm reduction community meetings for IDUs**

**Questionnaire**

**Please answer the following questions by writing down your answers, or by ticking (√) the appropriate box (). If you have trouble in answering a question, do not hesitate to ask the enumerator.**

| **A.** | **RESPONDENT PROFILE** | | |
| --- | --- | --- | --- |
| 1. | ID : | __________________________________________ | |
| 2. | Place of stay : | __________________________________________ | |
| 3. | Sex: |  Male 1  Female 2 | |
| 4. | Age : | ………… year | |
| 5. | Last degree of completed education: | | |
|  |  1. Not going to school  2. Primary School  3. Junior High School  4. Senior High School   5. Diploma  6. Bachelor Degree  7. Master Degree  8. Doctoral Degree | | |
| 6. | Current marital status: | | |
|  |  not yet married 0  married 1  divorced 2  widowed 3 | | |
| 7. | Number of children | ………… | |
| **B** | **OCCUPATION AND INCOME** | | |
| 8. | What is your current occupation?  (you may give more than one answer, proceed question 12 if you do not have a paid job) | | |
|  | ---------------------------------------------------------------------------------------------------------------------------- | | |
| 9. | What is your average monthly income?  (if you have more than one occupation, please state the total income)    **Rp** …………….…………………… | | |
| 10. | How many hours do you work per day? | | …… hours |
| 11. | How many days do you work per week? | | …… days |
| 12. | To fulfill your own monthly needs, do you also receive money from other people? Please state in the following table as well as the amount. | | |
|  | \| **No** \| **Receive money from** \| **Last month amount** \| \| --- \| --- \| --- \| \| **1** \| Parents \| Rp…………………………….. \| \| **2** \| Siblings \| Rp…………………………….. \| \| **3** \| Other relatives \| Rp…………………………….. \| \| **4** \| Children \| Rp…………………………….. \| \| **5** \| Friends \| Rp…………………………….. \| \| **6** \| Selling own goods \| Rp…………………………….. \| \| **7** \| Borrow from …. \| Rp…………………………….. \| \| **8** \| Others, please state………. \| Rp…………………………….. \| | | |
|  |  | | |
| 13. | Please state and detail your own monthly expenses | | |
|  | \| **No** \| **Expenses** \| **Last month amount** \| \| --- \| --- \| --- \| \| **1** \| House rent/mortgage \| Rp ………………………………………… \| \| **2** \| Electricity, water, telephone \| Rp ………………………………………… \| \| **3** \| Water \| Rp ………………………………………… \| \| **4** \| Telephone \| Rp ………………………………………… \| \| **5** \| Transport/gasoline \| Rp ………………………………………… \| \| **6** \| Cellphone credit \| Rp ………………………………………… \| \| **7** \| Food at home \| Rp ………………………………………… \| \| **8** \| Food out of home \| Rp ………………………………………… \| \| **9** \| Entertainment (i.e. snacks, cinema) \| Rp ………………………………………… \| \| **10** \| Cigarettes \| Rp ………………………………………… \| \| **11** \| Health/doctor fee \| Rp ………………………………………… \| \| **12** \| Medication \| Rp ………………………………………… \| \| **13** \| Savings \| Rp ………………………………………… \| \| **14** \| Others, please state……………………… \| Rp………………………………………….. \| | | |
|  |  | | |
| 14. | Who is currently staying with you?  **To fill the following tabel**:   - Please circle the number of the person who is currently living with you, if he/she is not on the list, please state on number 7-10 - Please state the occupation of the person (including housewife, students, or unemployed) and his/her income | | |
|  | \| 1. **Family member/**   **accompanying person** \| \| 1. **Occupation** \| 1. **Monthly income** \| 1. **Does this person accompany you to the health facility?** \| \| --- \| --- \| --- \| --- \| --- \| \| **1** \| Father \| ...... \| Rp........................ \| **** \| \| **2** \| Mother \| ...... \| Rp........................ \| **** \| \| **3** \| Older sibling \| ...... \| Rp........................ \| **** \| \| **4** \| Younger sibling \| ...... \| Rp........................ \| **** \| \| **5** \| Spouse \| ...... \| Rp........................ \| **** \| \| **6** \| Child \| ...... \| Rp........................ \| **** \| \| **7** \| ...... \| ...... \| Rp........................ \| **** \| \| **8** \| ...... \| ...... \| Rp........................ \| **** \| \| **9** \| ...... \| ...... \| Rp........................ \| **** \| \| **10** \| ...... \| ...... \| Rp........................ \| **** \| | | |

| **C** | **A VISIT TO THE FACILITY** |
| --- | --- |

| 15. | How do you reach the facility? | |
| --- | --- | --- |
|  |  1. On foot   2. Bicycle   3. Motorcycle   4. Motorcycle taxi   5. Car |  6. Public transport (car)   7. Taxi   8. Public transport (bus)   9. Others, please state………… |
| 16. | How long is your travel time to reach the facility? | …… minutes |
| 17. | On your visit to the facility, how much do you spent in average for: | |
|  | \| 1. Two way transport for yourself \| Rp................ \| \| --- \| --- \| \| 1. Two way transport for person(s) accompanying you \| Rp................ \| \| 1. Registration \| Rp................ \| \| 1. Medical treatment \| Rp................ \| \| 1. Materials received during meeting \| Rp................ \| \| 1. …… \| Rp................ \| \| 1. …… \| Rp................ \| | |

18. How much time do you spent in the facility? (including waiting time) ……..minutes

| **D** | **EFFECTIVENESS** |
| --- | --- |

19. What mark would you give the harm reduction community meeting from 1 up to 10 regarding the accessibility of the meeting? Please explain.

...........................................................................................................................................................................................................................................................................................................................................................................................................................................................................................................................................................................................................................

20. What mark would you give the harm reduction community meeting from 1 up to 10 regarding the clarity of the information? Please explain.

...........................................................................................................................................................................................................................................................................................................................................................................................................................................................................................................................................................................................................................

21. What mark would you give the harm reduction community meeting from 1 up to 10 regarding the duration of the meeting? Please explain.

...........................................................................................................................................................................................................................................................................................................................................................................................................................................................................................................................................................................................................................

22. What mark would you give the harm reduction community meeting from 1 up to 10 regarding the quality of the teacher? Please explain.

...........................................................................................................................................................................................................................................................................................................................................................................................................................................................................................................................................................................................................................

23. What did you learn in the harm reduction community meeting?

...........................................................................................................................................................................................................................................................................................................................................................................................................................................................................................................................................................................................................................

24. From 1 up to 10, how much did you feel like you possessed all the knowledge about HIV prevention before attending the meeting?

...........................................................................................................................................................................................................................................................................................................................................................................................................................................................................................................................................................................................................................

25. What are positive aspects of the harm reduction community meetings?

...........................................................................................................................................................................................................................................................................................................................................................................................................................................................................................................................................................................................................................

26. What do you think that could be improved of the harm reduction community meetings?

...........................................................................................................................................................................................................................................................................................................................................................................................................................................................................................................................................................................................................................

**Kuisioner**

**Jawab pertanyaan-pertanyaan di bawah ini dengan cara menuliskan jawaban, menceklis (√) di kotak (). Apabila Anda punya pertanyaan, jangan ragu untuk bertanya kepada enumerator.**

| **A.** | **PROFIL RESPONDEN** | | |
| --- | --- | --- | --- |
| 1. | NAMA : | __________________________________________ | |
| 2. | Tempat Tinggal : | __________________________________________ | |
| 3. | Jenis Kelamin : |  Pria 1  Wanita 2 | |
| 4. | Usia : | ………… tahun | |
| 5. | Pendidikan terakhir: | | |
|  |  1. Tidak bersekolah  2. Sekolah Dasar  3. Sekolah Menengah Pertama  4. Sekolah Menengah Atas  5. Diploma  6. Sarjana  7. Master  8. Doktor | | |
| 6. | Status Pernikahan: | | |
|  |  belum menikah 0  menikah 1  bercerai 2  janda 3 | | |
| 7. | Jumlah anak | ………… | |
| **B** | **PEKERJAAN DAN PENDAPATAN** | | |
| 8. | Apa pekerjaan Anda saat ini? (boleh menjawab lebih dari satu pekerjaan, langsung ke pertanyaan nomor 12 apabila pekerjaan adalah pekerjaan tidak dibayar) | | |
|  | ---------------------------------------------------------------------------------------------------------------------------- | | |
| 9. | Berapa pendapatan rata-rata Anda perbulan? (apabila Anda mempunyai lebih dari satu pekerjaan, sebutkan pendapatan total)    **Rp** …………….…………………… | | |
| 10. | Berapa jam Anda bekerja setiap hari? | | …… jam |
| 11. | Berapa hari Anda bekerja setiap minggu? | | …… hari |
| 12. | Untuk memenuhi kebutuhan Anda perbulan, apakah Anda menerima uang dari pihak lain? Sebutkan di table di bawah ini sesuai dengan jumlahnya. | | |
|  | \| **No** \| **Mendapatkan uang dari** \| **Jumlah bulan kemarin** \| \| --- \| --- \| --- \| \| **1** \| Orang Tua \| Rp…………………………….. \| \| **2** \| Kakak/Adik \| Rp…………………………….. \| \| **3** \| Saudara Lain \| Rp…………………………….. \| \| **4** \| Anak-anak \| Rp…………………………….. \| \| **5** \| Teman-teman \| Rp…………………………….. \| \| **6** \| Menjual barang pribadi \| Rp…………………………….. \| \| **7** \| Meminjam dari …. \| Rp…………………………….. \| \| **8** \| Lain-lain, sebutkan………. \| Rp…………………………….. \| | | |
| 13. | Sebutkan pengeluaran Anda setiap bulannya | | |
|  | \| **No** \| **Pengeluaran** \| **Jumlah bulan kemarin** \| \| --- \| --- \| --- \| \| **1** \| Sewa rumah/mortgage \| Rp ………………………………………… \| \| **2** \| Listrik \| Rp ………………………………………… \| \| **3** \| Air \| Rp ………………………………………… \| \| **4** \| Telepon \| Rp ………………………………………… \| \| **5** \| Transportasi/bensin \| Rp ………………………………………… \| \| **6** \| Pulsa \| Rp ………………………………………… \| \| **7** \| Makanan di rumah \| Rp ………………………………………… \| \| **8** \| Makanan di luar rumah \| Rp ………………………………………… \| \| **9** \| Hiburan (i.e. snacks, bioskop) \| Rp ………………………………………… \| \| **10** \| Rokok \| Rp ………………………………………… \| \| **11** \| Kesehatan \| Rp ………………………………………… \| \| **12** \| Obat-obatan \| Rp ………………………………………… \| \| **13** \| Tabungan \| Rp ………………………………………… \| \| **14** \| Lain-lain, sebutkan……………………… \| Rp………………………………………….. \| | | |
|  |  | | |
| 14. | Siapa yang saat ini tinggal bersama Anda?  **Untuk mengisi tabel berikut ini**:   - Bulatkan jumlah orang yang saat ini tinggal bersama Anda, jika dia tidak ada di daftar, sebutkan di nomor 7-10 - Sebutkan pekerjaan orang tersebut (termasuk ibu rumah tangga, pelajar, atau pengangguran) dan pendapatan mereka | | |
|  | \| 1. Anggota keluarga/   Orang yang tinggal bersama \| \| 1. Pekerjaan \| 1. Pendapatan perbulan \| 1. Apakah orang tersebut menemani Anda ke fasilitas kesehatan? \| \| --- \| --- \| --- \| --- \| --- \| \| 1 \| Ayah \| ...... \| Rp........................ \| **** \| \| 2 \| Ibu \| ...... \| Rp........................ \| **** \| \| 3 \| Kakak \| ...... \| Rp........................ \| **** \| \| 4 \| Adik \| ...... \| Rp........................ \| **** \| \| 5 \| Pasangan \| ...... \| Rp........................ \| **** \| \| 6 \| Anak \| ...... \| Rp........................ \| **** \| \| 7 \| ...... \| ...... \| Rp........................ \| **** \| \| 8 \| ...... \| ...... \| Rp........................ \| **** \| \| 9 \| ...... \| ...... \| Rp........................ \| **** \| \| 10 \| ...... \| ...... \| Rp........................ \| **** \| | | |

| 15. | Bagaimana Anda pergi ke fasilitas kesehatan? | |
| --- | --- | --- |
|  |  1. Jalan kaki   2. Sepeda   3. Sepeda motor   4. Ojek   5. Mobil |  6. Transportasi umum (mobil)   7. Taxi   8. Transportasi umum (bus)   9. Lain-lain,sebutkan………… |
| 16. | Berapa lama perjalanan Anda ke pertemuan harm reduction? | …… menit |
| 17. | Dalam kunjungan Anda ke pertemuan harm reduction, berapa banyak pengeluaran rata-rata Anda untuk: | |
|  | \| 1. Transportasi pulang pergi untuk Anda sendiri \| Rp................ \| \| --- \| --- \| \| 1. Transportasi pulang pergi untuk orang-orang yang menemani Anda \| Rp................ \| \| 1. Registrasi \| Rp................ \| \| 1. Perawatan medis \| Rp................ \| \| 1. Item yang diterima di rapat \| Rp................ \| \| 1. …… \| Rp................ \| | |

18. Berapa lama waktu yang Anda habiskan di pertemuan harm reduction? (termasuk waktu menunggu) ……..menit

19. Dari 1 sampai 10, Berapa nilai yang akan Anda berikan kepada pertemuan harm reduction mengenai aksesibilitas dari rapat penyuluhan? Tolong jelaskan ...............................................................................................................................................................................................…………………………………………………………………………………………………

20. Dari 1 sampai 10, Berapa nilai yang akan Anda berikan kepada pertemuan harm reduction mengenai kejelasan informasi? Tolong jelaskan.

.....................................................................................................................................................................................................................................................................................................................................................

21. Dari 1 sampai 10, Berapa nilai yang akan Anda berikan kepada pertemuan harm reduction mengenai durasi pertemuan? Tolong jelaskan.

....................................................................................................................................................................................................................................................................................................................................................

22. Dari 1 sampai 10, Berapa nilai yang akan Anda berikan kepada pertemuan harm reduction mengenai kualitas pengajar? Tolong jelaskan

....................................................................................................................................................................................................................................................................................................................................................

23. Apa saja yang Anda pelajari di pertemuan harm reduction ?

....................................................................................................................................................................................................................................................................................................................................................

24. Dari skala 1 sampai 10, seberapa banyak Anda merasa mengetahui tentang pencegahan HIV sebelum menghadiri pertemuan ini?

..................................................................................................................................................................................................................................................................................................................................................

25. Apa saja aspek-aspek positif dari pertemuan harm reduction?

....................................................................................................................................................................................................................................................................................................................................................

26. Menurut Anda, apa saja yang bias ditingkatkan dari pertemuan harm reduction?

....................................................................................................................................................................................................................................................................................................................................................

**IEC at maternal & child health posts**

**Questionnaire**

**Please answer the following questions by writing down your answers, or by ticking (√) the appropriate box (). If you have trouble in answering a question, do not hesitate to ask the enumerator.**

| **A.** | **RESPONDENT PROFILE** | | |
| --- | --- | --- | --- |
| 1. | ID : | __________________________________________ | |
| 2. | Place of stay : | __________________________________________ | |
| 3. | Sex: |  Male 1  Female 2 | |
| 4. | Age : | ………… year | |
| 5. | Last degree of completed education: | | |
|  |  1. Not going to school  2. Primary School  3. Junior High School  4. Senior High School   5. Diploma  6. Bachelor Degree  7. Master Degree  8. Doctoral Degree | | |
| 6. | Current marital status: | | |
|  |  not yet married 0  married 1  divorced 2  widowed 3 | | |
| 7. | Number of children | ………… | |
| **B** | **OCCUPATION AND INCOME** | | |
| 8. | What is your current occupation (may give more than one answer, proceed no 12 it non paid job) | | |
|  | ---------------------------------------------------------------------------------------------------------------------------- | | |
| 9. | What is your average monthly income?  (if you have more than one occupation, please state the total income)    **Rp** …………….…………………… | | |
| 10. | How many hours do you work per day? | | …… hours |
| 11. | How many days do you work per week? | | …… days |
| 12. | To fulfill your own monthly needs, do you also receive money from other people? Please state in the following table as well as the amount. | | |
|  | \| **No** \| **Receive money from** \| **Last month amount** \| \| --- \| --- \| --- \| \| **1** \| Parents \| Rp…………………………….. \| \| **2** \| Siblings \| Rp…………………………….. \| \| **3** \| Other relatives \| Rp…………………………….. \| \| **4** \| Children \| Rp…………………………….. \| \| **5** \| Friends \| Rp…………………………….. \| \| **6** \| Selling own goods \| Rp…………………………….. \| \| **7** \| Borrow from …. \| Rp…………………………….. \| \| **8** \| Others, please state………. \| Rp…………………………….. \| | | |
|  |  | | |
| 13. | Please state and detail your own monthly expenses | | |
|  | \| **No** \| **Expenses** \| **Last month amount** \| \| --- \| --- \| --- \| \| **1** \| House rent/mortgage \| Rp ………………………………………… \| \| **2** \| Electricity, water, telephone \| Rp ………………………………………… \| \| **3** \| Water \| Rp ………………………………………… \| \| **4** \| Telephone \| Rp ………………………………………… \| \| **5** \| Transport/gasoline \| Rp ………………………………………… \| \| **6** \| Cellphone credit \| Rp ………………………………………… \| \| **7** \| Food at home \| Rp ………………………………………… \| \| **8** \| Food out of home \| Rp ………………………………………… \| \| **9** \| Entertainment (i.e. snacks, cinema) \| Rp ………………………………………… \| \| **10** \| Cigarettes \| Rp ………………………………………… \| \| **11** \| Health/doctor fee \| Rp ………………………………………… \| \| **12** \| Medication \| Rp ………………………………………… \| \| **13** \| Savings \| Rp ………………………………………… \| \| **14** \| Others, please state……………………… \| Rp………………………………………….. \| | | |
|  |  | | |
| 14. | Who is currently staying with you?  **To fill the following tabel**:   - Please circle the number of the person who is currently living with you, if he/she is not on the list, please state on number 7-10 - Please state the occupation of the person (including housewife, students, or unemployed) and his/her income | | |
|  | \| 1. Family member/   accompanying person \| \| 1. Occupation \| 1. Monthly income \| 1. Does this person accompany you to the health facility? \| \| --- \| --- \| --- \| --- \| --- \| \| 1 \| Father \| ...... \| Rp........................ \| **** \| \| 2 \| Mother \| ...... \| Rp........................ \| **** \| \| 3 \| Older sibling \| ...... \| Rp........................ \| **** \| \| 4 \| Younger sibling \| ...... \| Rp........................ \| **** \| \| 5 \| Spouse \| ...... \| Rp........................ \| **** \| \| 6 \| Child \| ...... \| Rp........................ \| **** \| \| 7 \| ...... \| ...... \| Rp........................ \| **** \| \| 8 \| ...... \| ...... \| Rp........................ \| **** \| \| 9 \| ...... \| ...... \| Rp........................ \| **** \| \| 10 \| ...... \| ...... \| Rp........................ \| **** \| | | |

| 15. | How do you reach the facility? | |
| --- | --- | --- |
|  |  1. On foot   2. Bicycle   3. Motorcycle   4. Motorcycle taxi   5. Car |  6. Public transport (car)   7. Taxi   8. Public transport (bus)   9. Others, please state………… |
| 16. | How long is your travel time to reach the facility? | …… minutes  How far is your travel distance to reach the posyandu? |
| 17. | On your visit to the facility, how much do you spent in average for: | |
|  | \| 1. Two way transport for yourself \| Rp................ \| \| --- \| --- \| \| 1. Two way transport for person(s) accompanying you \| Rp................ \| \| 1. Registration \| Rp................ \| \| 1. Medical treatment \| Rp................ \| \|  \| Rp................ \| \|  \| Rp................ \| \|  \| Rp................ \| | |

C. HIV IEC MEETING

For what primary purpose did you visit the posyandu today?

- - For the HIV IEC meeting
  - Other:.............................................................................................................................................................................................................................................................................................................................................................................................................................................................................................................

How did you hear about the HIV IEC meeting?

- - I heard it today when visiting the posyandu
  - I heard it from friends or relatives
  - I heard it from family
  - I heard it from the health cadre
  - I heard it when I visited the posyandu previously
  - Other:.............................................................................................................................................................................................................................................................................................................................................................................................................................................................................................................

Was this your first time visiting the HIV IEC meeting?

- - Yes
  - No

IF NO, how often do you visit an HIV IEC meeting?

**Please give an indication per year*

......................................................................................................................................................................................................... .........................................................................................................................................................................................................

.........................................................................................................................................................................................................

What mark would you give the HIV IEC meeting from 1 up to 10 regarding the accessibility of the meeting? Please explain.

......................................................................................................................................................................................................... ......................................................................................................................................................................................................... .........................................................................................................................................................................................................

What mark would you give the HIV IEC meeting from 1 up to 10 regarding the clarity of the information? Please explain.

......................................................................................................................................................................................................... ......................................................................................................................................................................................................... .........................................................................................................................................................................................................

What mark would you give the HIV IEC meeting from 1 up to 10 regarding the duration of the meeting? Please explain.

......................................................................................................................................................................................................... ......................................................................................................................................................................................................... .........................................................................................................................................................................................................

What mark would you give the HIV IEC meeting from 1 up to 10 regarding the quality of the teacher? Please explain.

......................................................................................................................................................................................................... ......................................................................................................................................................................................................... .........................................................................................................................................................................................................

What did you learn in the HIV IEC meeting?

......................................................................................................................................................................................................... ......................................................................................................................................................................................................... .........................................................................................................................................................................................................

From 1 up to 10, how much did you feel like you possessed all the knowledge about HIV prevention before attending the meeting?

......................................................................................................................................................................................................... ......................................................................................................................................................................................................... .........................................................................................................................................................................................................

From 1 up to 10, how much do you now feel like you possess all the knowledge about HIV prevention after attending the meeting?

......................................................................................................................................................................................................... ......................................................................................................................................................................................................... .........................................................................................................................................................................................................

What are positive aspects of the HIV IEC meetings?

......................................................................................................................................................................................................... ......................................................................................................................................................................................................... .........................................................................................................................................................................................................

What do you think that could be improved of the HIV IEC meetings?

......................................................................................................................................................................................................... ......................................................................................................................................................................................................... ........................................................................................................................................................................................................

**Kuisioner**

**Jawab pertanyaan-pertanyaan di bawah ini dengan cara menuliskan jawaban, menceklis (√) di kotak (). Apabila Anda punya pertanyaan, jangan ragu untuk bertanya kepada enumerator.**

1. **PROFIL RESPONDEN**
2. Nama: ………………………………………………………...
3. Tempat Tinggal: ………………………………………………………...
4. Jenis Kelamin:  Pria 1  Wanita 2
5. Usia: ………… tahun
6. Pendidikan terakhir:

 1. Tidak bersekolah  2. Sekolah Dasar  3. Sekolah Menengah Pertama

 4. Sekolah Menengah Atas  5. Diploma  6. Sarjana  7. Master  8. Doktor

1. Status Pernikahan

 belum menikah 0  menikah 1  bercerai 2  janda 3

1. Jumlah anak …………
2. **PEKERJAAN DAN PENDAPATAN**
3. Apa pekerjaan Anda saat ini? (boleh menjawab lebih dari satu pekerjaan, langsung ke pertanyaan nomor 12 apabila pekerjaan adalah pekerjaan tidak dibayar)

…………………………………………………………………………………………………...

…………………………………………………………………………………………………...

1. Berapa pendapatan rata-rata Anda perbulan? (apabila Anda mempunyai lebih dari satu pekerjaan, sebutkan pendapatan total)

**Rp** …………….……………………

1. Berapa jam Anda bekerja setiap hari?

…………… jam

1. Berapa hari Anda bekerja setiap minggu?

……...……. hari

1. Untuk memenuhi kebutuhan Anda perbulan, apakah Anda menerima uang dari pihak lain? Sebutkan di table di bawah ini sesuai dengan jumlahnya.

| **No** | **Mendapatkan uang dari** | **Jumlah bulan kemarin** |
| --- | --- | --- |
| 1 | Orang Tua | Rp…………………………….. |
| 2 | Kakak/Adik | Rp…………………………….. |
| 3 | Saudara Lain | Rp…………………………….. |
| 4 | Anak-anak | Rp…………………………….. |
| 5 | Teman-teman | Rp…………………………….. |
| 6 | Menjual barang pribadi | Rp…………………………….. |
| 7 | Meminjam dari …. | Rp…………………………….. |
| 8 | Lain-lain, sebutkan………. | Rp…………………………….. |

1. Sebutkan pengeluaran Anda setiap bulannya

| **No** | **Pengeluaran** | **Jumlah bulan kemarin** |
| --- | --- | --- |
| 1 | Sewa rumah/mortgage | Rp ………………………………………… |
| 2 | Listrik | Rp ………………………………………… |
| 3 | Air | Rp ………………………………………… |
| 4 | Telepon | Rp ………………………………………… |
| 5 | Transportasi/bensin | Rp ………………………………………… |
| 6 | Pulsa | Rp ………………………………………… |
| 7 | Makanan di rumah | Rp ………………………………………… |
| 8 | Makanan di luar rumah | Rp ………………………………………… |
| 9 | Hiburan (i.e. snacks, bioskop) | Rp ………………………………………… |
| 10 | Rokok | Rp ………………………………………… |
| 11 | Kesehatan | Rp ………………………………………… |
| 12 | Obat-obatan | Rp ………………………………………… |
| 13 | Tabungan | Rp ………………………………………… |
| 14 | Lain-lain, sebutkan……………………… | Rp………………………………………….. |

1. Siapa yang saat ini tinggal bersama Anda?

**Untuk mengisi tabel berikut ini**:

- Bulatkan jumlah orang yang saat ini tinggal bersama Anda, jika dia tidak ada di daftar, sebutkan di nomor 7-10
- Sebutkan pekerjaan orang tersebut (termasuk ibu rumah tangga, pelajar, atau pengangguran) dan pendapatan mereka

| Anggota keluarga/  Orang yang tinggal bersama | | Pekerjaan | Pendapatan perbulan | Apakah orang tersebut menemani Anda ke posyandu? |
| --- | --- | --- | --- | --- |
| 1 | Ayah | ...... | Rp........................ | **** |
| 2 | Ibu | ...... | Rp........................ | **** |
| 3 | Kakak | ...... | Rp........................ | **** |
| 4 | Adik | ...... | Rp........................ | **** |
| 5 | Pasangan | ...... | Rp........................ | **** |
| 6 | Anak | ...... | Rp........................ | **** |
| 7 | ...... | ...... | Rp........................ | **** |
| 8 | ...... | ...... | Rp........................ | **** |
| 9 | ...... | ...... | Rp........................ | **** |
| 10 | ...... | ...... | Rp........................ | **** |

1. Bagaimana Anda pergi ke posyandu?

 1. Jalan kaki  6. Transportasi umum (mobil)

 2. Sepeda  7. Taxi

 3. Sepeda motor  8. Transportasi umum (bus)

 4. Ojek  9. Lain-lain, sebutkan ……………

 5. Mobil

1. Berapa lama perjalanan Anda ke posyandu?

……….. menit

1. Berapa lama perjalanan Anda ke HIV IEC?

……….. menit

1. Dalam kunjungan Anda ke posyandu, berapa banyak pengeluaran rata-rata Anda untuk:

| 1. Transportasi pulang pergi untuk Anda sendiri | Rp................ |
| --- | --- |
| 1. Transportasi pulang pergi untuk orang-orang yang menemani Anda | Rp................ |
| 1. Registrasi | Rp................ |
| 1. Perawatan medis | Rp................ |
| 1. Pemeriksaan laboratorium | Rp................ |
| 1. ……. | Rp................ |
| 1. ……. | Rp................ |
| 1. …… | Rp................ |

1. Berapa lama waktu yang Anda habiskan di posyandu? (termasuk waktu menunggu)

……….. menit

1. **Pertemuan HIV IEC**
2. Apa tujuan utama Anda dating ke Posyandu hari ini?

- Untuk pertemuan HIV IEC
- Lainnya:................................................................................................................................................................................................................................................................................................................................................................................................................

1. Bagaimana Anda mengetahui tentang pertemuan HIV IEC?

- Saya tahu hari ini ketika dating ke Posyandu
- Saya tahu dari teman atau saudara
- Saya tahu dari keluarga
- Saya tahu dari kader kesehatan
- Saya tahu ketika saya datang ke Posyandu sebelumnya
- Lainnya:..............................................................................................................................................................................................................................................................................................................................................................................................................

1. Apakah ini pertama kalinya Anda mendatangi pertemuan HIV IEC?

- Iya
- Tidak

**JIKA TIDAK,** seberapa sering Anda datang ke pertemuan HIV IEC?

**Tolong jawab jumlah dalam setahun*

.....................................................................................................................................................................................................................................................................................................................................................................................................................................................................

1. Dari 1 sampai 10, Berapa nilai yang akan Anda berikan kepada pertemuan HIV IEC mengenai aksesibilitas dari rapat penyuluhan? Tolong jelaskan.

.....................................................................................................................................................................................................................................................................................................................................................................................................................................................................

1. Dari 1 sampai 10, Berapa nilai yang akan Anda berikan kepada pertemuan HIV IEC mengenai kejelasan informasi? Tolong jelaskan.

.....................................................................................................................................................................................................................................................................................................................................................................................................................................................................

1. Dari 1 sampai 10, Berapa nilai yang akan Anda berikan kepada pertemuan HIV IEC mengenai durasi pertemuan? Tolong jelaskan.

.....................................................................................................................................................................................................................................................................................................................................................................................................................................................................

1. Dari 1 sampai 10, Berapa nilai yang akan Anda berikan kepada pertemuan HIV IEC mengenai kualitas pengajar? Tolong jelaskan.

.....................................................................................................................................................................................................................................................................................................................................................................................................................................................................

1. Apa saja yang Anda pelajari di pertemuan HIV IEC?

.....................................................................................................................................................................................................................................................................................................................................................................................................................................................................

1. Dari skala 1 sampai 10, seberapa banyak Anda merasa mengetahui tentang pencegahan HIV sebelum menghadiri pertemuan ini?

.....................................................................................................................................................................................................................................................................................................................................................................................................................................................................

1. Dari skala 1 sampai 10, seberapa banyak Anda merasa mengetahui tentang pencegahan HIV setelah menghadiri pertemuan ini?

.....................................................................................................................................................................................................................................................................................................................................................................................................................................................................

1. Apa saja aspek-aspek positif dari pertemuan HIV IEC?

.....................................................................................................................................................................................................................................................................................................................................................................................................................................................................

1. Menurut Anda, apa saja yang bias ditingkatkan dari pertemuan HIV IEC?

.....................................................................................................................................................................................................................................................................................................................................................................................................................................................................

**Terima kasih banyak!**
